# Two cases of rare HHV8-driven intravascular lymphoma with synchronous Kaposi sarcoma, both diagnosed at autopsy in renal transplant recipients.

**DOI:** 10.4322/acr.2020.206

**Published:** 2020-11-20

**Authors:** Paida Gwiti, Megan Jenkins, Judit Sutak, Zsombor Melegh

**Affiliations:** 1 Peterborough City Hospital, Department of Cellular Pathology, Peterborough, UK; 2 Royal Liverpool University Hospital, Forensic Pathology Unit, Liverpool, UK; 3 Department of Cellular Pathology, North Bristol NHS Trust, Bristol, UK

**Keywords:** Herpesvirus 8, Human, Sarcoma, Kaposi, Autopsy, Transplantation, Lymphoproliferative disorder, Intravascular lymphoma

## Abstract

We present the first report of two rare yet remarkably similar autopsy cases of Kaposi sarcoma (KS) and intravascular human herpesvirus 8 (HHV8) positive lymphoproliferative disorder in renal transplant patients. It is well established that HHV8 infection causes Kaposi sarcoma (KS). More recently, it is recognized that HHV8 is also related to several lymphoproliferative conditions. These are poorly characterized and often difficult to diagnose. In both cases described herein, the diagnoses of multifocal hepatic KS and intravascular HHV8 positive (EBV negative) systemic diffuse large B-cell lymphoma, NOS were made at autopsy. Given the findings we describe in cases with fatal outcomes, we discuss the implications of HHV8 screening in solid allograft recipients.

## INTRODUCTION

Human herpesvirus 8 (HHV8), also known as Kaposi’s Sarcoma-associated Herpes Virus, is a gamma-2 herpesvirus and is related to Epstein-Barr virus (EBV), a member of the gamma-1 subfamily; both viruses are known to be oncogenic.^1^ HHV8 can infect lymphoid and endothelial cells and is known to drive Kaposi sarcoma (KS) and lymphoid neoplasms, most often in the setting of concurrent human immunodeficiency virus (HIV) infection.^2^ ^,^ ^3^ Host factors seem to influence pathogenesis, since HHV8 seroprevalence is relatively common in contrast to the rarity of HHV8-associated disorders.^4^

We describe two remarkably similar autopsy cases that were performed within a 15-month interval in the UK. In each case, a Coroner’s autopsy was requested for a renal transplant recipient. Common autopsy findings include multifocal hepatic KS and intravascular HHV8-associated diffuse large B-cell lymphoma, not otherwise specified (DLBCL, NOS).

The atypical clinical presentation and pathologic features of these rare cases demonstrate the challenges in diagnosis, classification and management of HHV8-driven malignancies.

## AUTOPSY CASE 1

**Figure 1:**
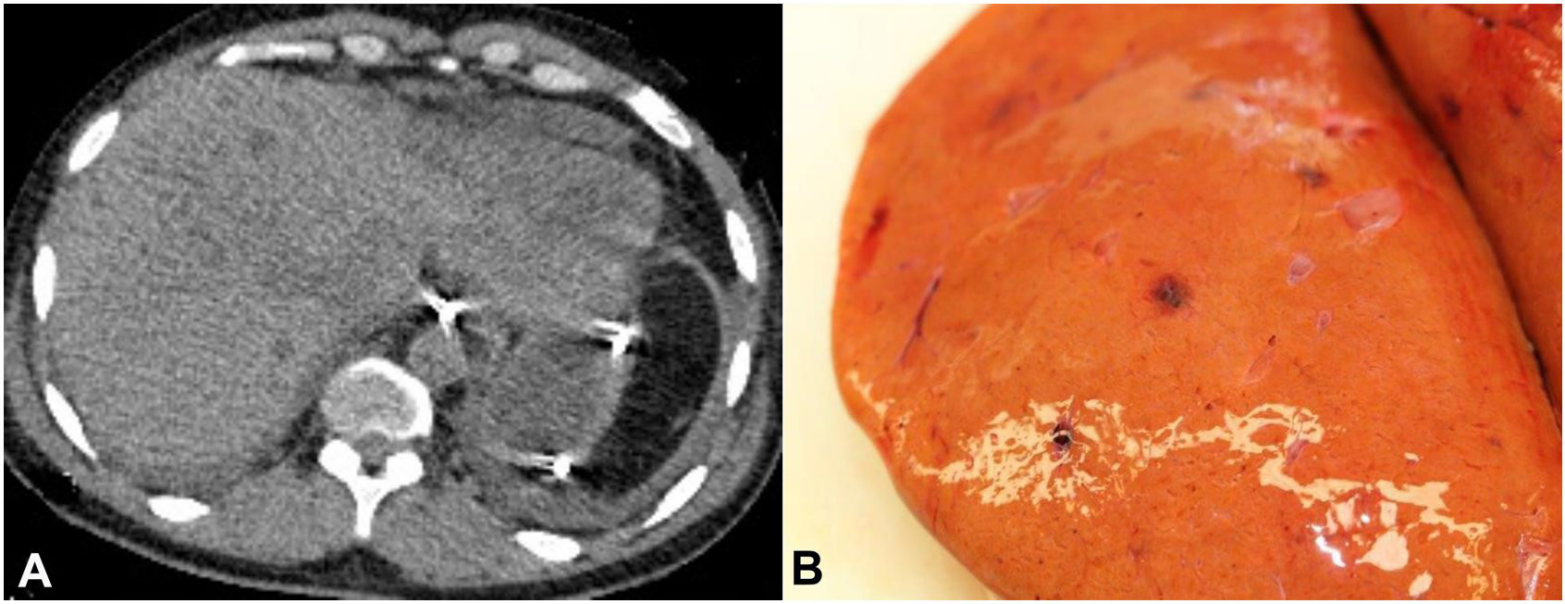
**A –** Antemortem CT scan of the abdomen showed innumerable low attenuation lesions throughout the liver, **B –** At autopsy, multiple hemorrhagic foci were observed throughout the liver.

A 37-year-old man presented to hospital with fever and elevated inflammatory markers. He had been diagnosed with pneumococcal septicemia five years previously, which was complicated by post-streptococcal glomerulonephritis and subsequent end-stage renal failure requiring renal transplant. At the time of admission, he was prescribed tacrolimus, 8mg *bd*, and prednisolone, 15mg *od*. He was admitted to hospital and treated empirically with antibiotics and Amphotericin B for a suspected infection. His condition deteriorated and he was transferred to the intensive care unit for multi-organ support. A CT scan revealed multiple, solid liver lesions suggestive of a fungal infection or lymphoma (Figure 1A). Soon after a liver biopsy he rapidly developed cardiovascular instability and died.

### Autopsy

A 2,000ml hemoperitoneum was found and the liver contained multiple haemorrhagic foci (Figure 1B). All other major organ systems appeared normal and there was no evidence of lymphadenopathy.

### Histology

**Figure 2:**
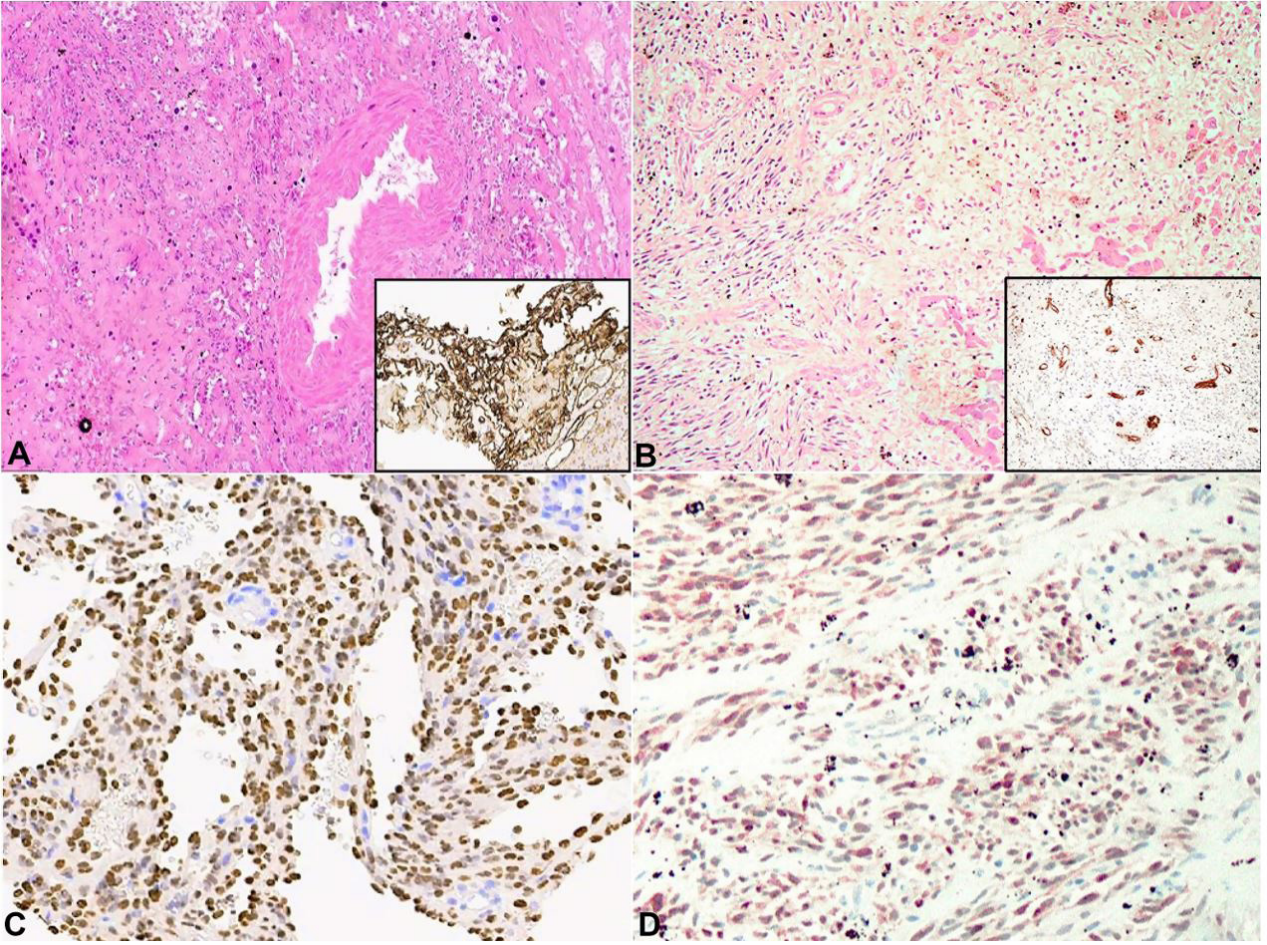
Comparison between Case 1, left column, and Case 2, right column. **A and B –** autopsy liver showing atypical spindle cell proliferation (H&E, 20X), with CD31 positivity in antemortem liver biopsy (a, insert) and post-mortem liver sample (b, insert), **C and D –** HHV8 positivity is observed in both cases.

The antemortem liver biopsy demonstrated a malignant vasoformative neoplasm comprising spindled cells with focal solid growth pattern around the portal tracts. The spindle component expressed CD31 and HHV8 (Figure 2A, 2C). Post-mortem liver histopathology showed a similar proliferation surrounding multiple, variably sized, blood-filled spaces. The features are those of a hepatic KS and explain the radiological finding of multiple liver lesions.

**Figure 4:**
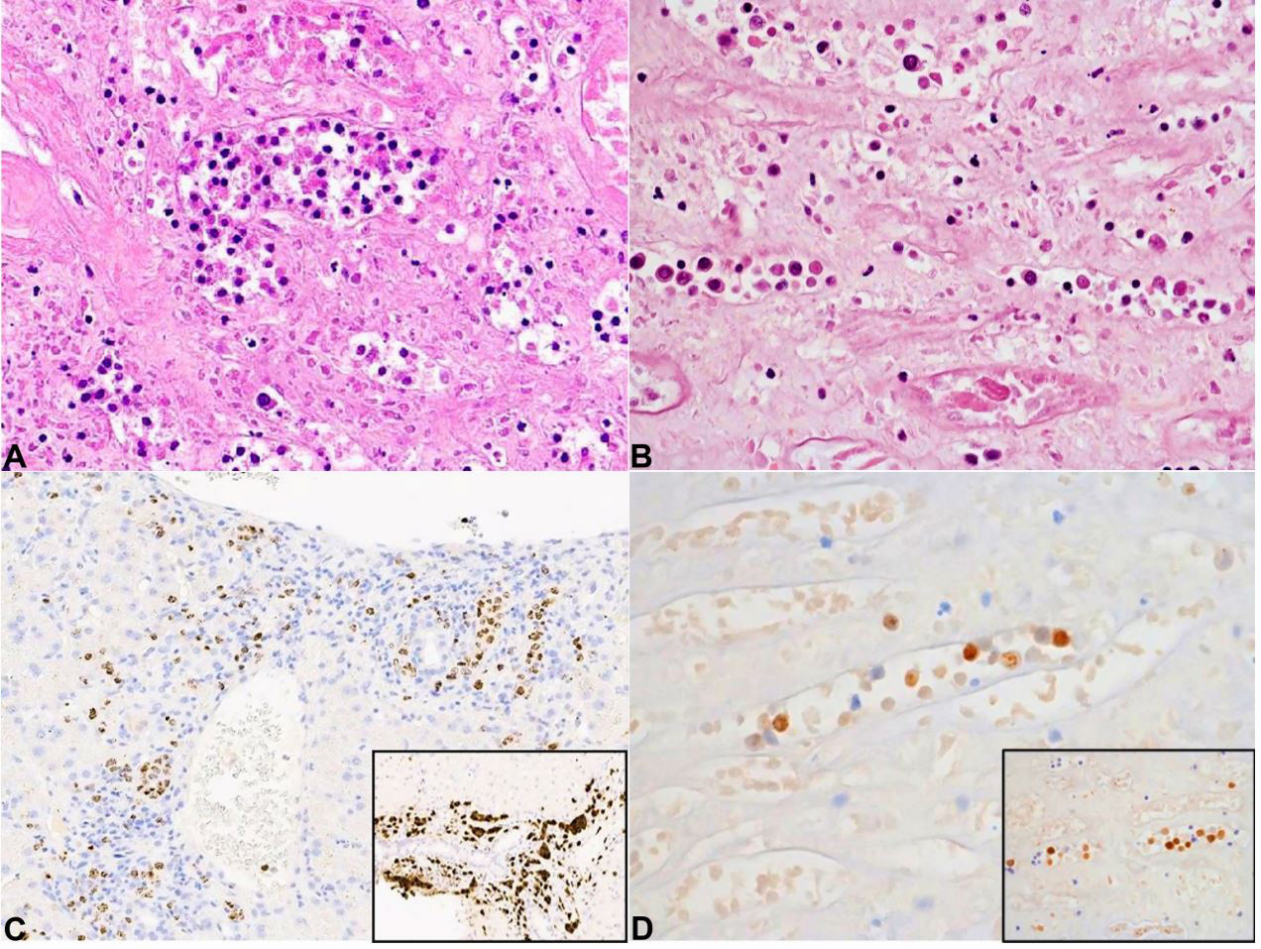
Comparison between Case 1, left column, and Case 2, right column. **A** and **B –** autopsy samples showing intravascular lymphoid proliferation (H&E, 20X), **C –** HHV8 positive intravascular large B-cell lymphoma present in antemortem liver biopsy; and **D –** post-mortem kidney, both with MUM1 positivity (insert).

The antemortem liver biopsy also showed atypical large lymphoid cells in portal tract vessels and hepatic sinusoids (Figure 4A, 4C). These neoplastic cells had plasmablastic morphology, with rounded nuclei, conspicuous nucleoli and moderate eosinophilic cytoplasm. Apoptotic debris and mitotic figures were easily identified. The neoplastic blasts expressed CD45, HHV8 (stippled nuclear positivity), MUM1, OCT2 and BOB1. Ki67 proliferation index was 80%. CD20, CD2, CD3, CD4, CD5, CD7, CD8, CD34, ALK, CD138, PAX-5, CD56 were negative. Epstein-Barr encoding region (EBER) *in situ* hybridization was negative.

The post-mortem liver biopsy showed the same neoplastic lymphoid blasts to those seen in the antemortem biopsy. There were arranged in a similar intravascular and sinusoidal distribution. HHV8 positive lymphoid blasts were also found in small vessels in the post-mortem lung and kidney. This was interpreted as systemic involvement by an intravascular HHV8 positive DLBCL, NOS.

## AUTOPSY CASE 2

A 42-year-old male who had previously undergone renal transplantation for hypertensive renal disease developed features of acute rejection. He was initially managed with anti-thymocyte globulin immunosuppressive therapy but developed HHV8 viremia. An infected donor kidney was considered the most likely source of infection; the recipient of the second donor kidney was known to have Kaposi sarcoma. The patient developed septic shock with an associated pancytopenia despite Ganciclovir therapy. He died of multiorgan failure.

### Autopsy

**Figure 3:**
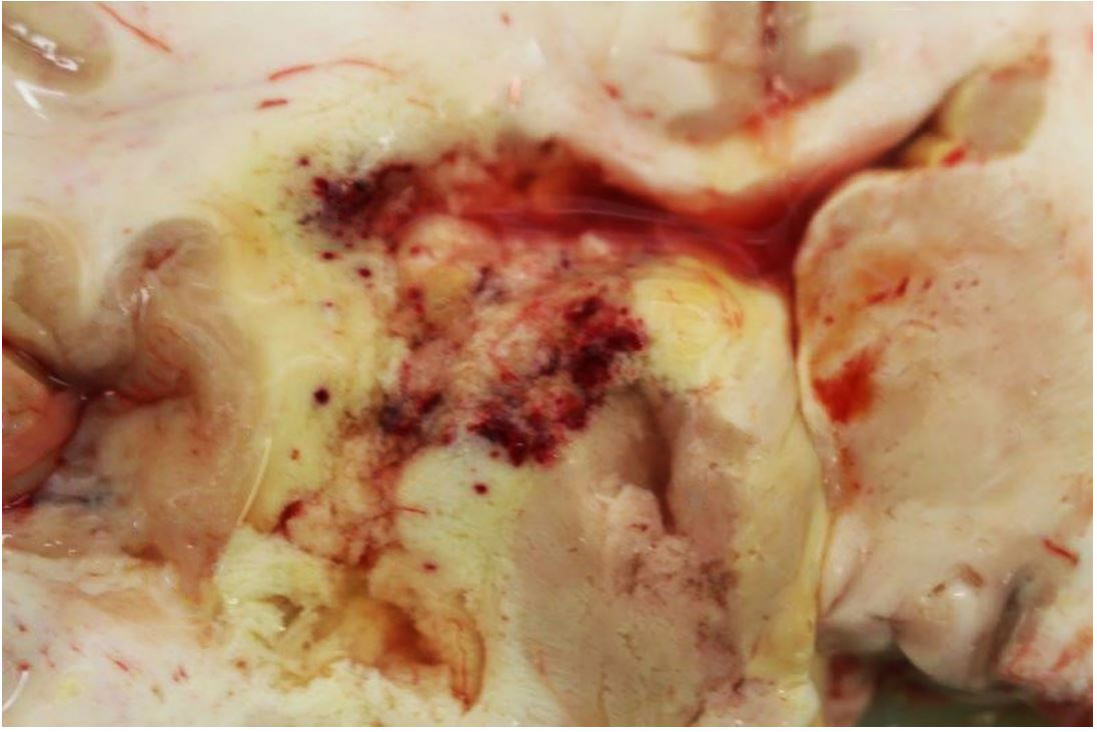
Gross view of the brain showing multiple areas of hemorrhagic infarction.

Post-mortem examination revealed areas of brain infarction (Figure 3); irregular, yellow nodules were observed throughout the right lung and multiple hemorrhagic foci were identified throughout the liver. Again, there was no evidence of lymphadenopathy.

### Histology

The post-mortem liver histology demonstrated multiple vasoformative spindle cell proliferations expressing HHV8 positivity (Figure 22D). A similar HHV8 positive spindle cell lesion was also identified in the lung. A diagnosis of multifocal KS was made. The histology of the parenchymal organs and spleen demonstrated intravascular lymphoid blasts with plasmablastic morphology and immunophenotype (Figure 44D).

**Figure 5:**
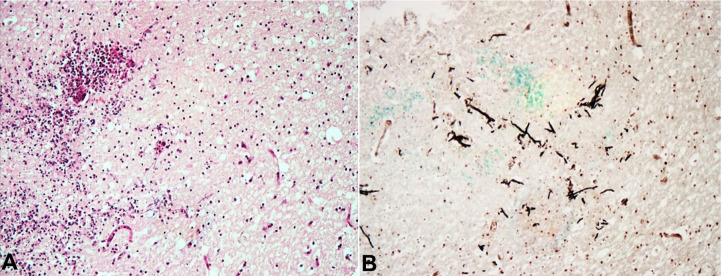
Photomicrographs of the brain demonstrating: **A –** inflamed brain tissue (H&E, 10X); **B –** fungal organisms with an acute angle of hyphal division, consistent with mucormycosis (Grocott, 10X).

Similar to Case 1, the blasts expressed CD45, HHV8, MUM1, OCT2 and BOB1. Ki67 proliferation index was 70%. CD3, CD20, PAX5 and CD138 were negative, as was EBER *in situ* hybridization. Once again, a diagnosis of intravascular HHV8 positive DLBCL, NOS was made. In addition, sections taken from those abnormal areas of brain and lung revealed fungal organisms consistent with systemic mucormycosis infection (Figure 5).

## DISCUSSION

Both cases demonstrate a plethora of rare immunosuppression-related (post-transplant) pathologies. At autopsy, both patients were diagnosed with multifocal hepatic KS, a vascular tumor that is observed in four distinct clinical settings.^5^ The ‘classical’ form primarily affects men of Ashkenazic Jewish or Mediterranean background, presenting with skin lesion that follow an indolent clinical course. The ‘African endemic’ form of the disease commonly affects Africans, presenting with lymphadenopathy that follows a more aggressive clinical course. The ‘AIDS associated’ form is observed amongst those infected by HIV and is the most aggressive of all KS subtypes. Our cases fall into the category of ‘immunosuppression-related’, an aggressive form of the disease that often presents in recipients of kidney or liver allografts and typically involves lymph nodes and visceral organs, occasionally with concurrent mucocutaneous involvement.^5^ In the absence of skin lesions, diagnosis is often made at autopsy due to the lack of associated symptoms.^6^ Across all subtypes, the etiologic agent is HHV8, either by activation of HHV8 infection in a seropositive recipient, or by transmission of the virus from a seropositive donor. Studies investigating HHV8 infection amongst French cohorts in the post-transplant setting have found that, where the donor was HHV8 positive, around one third of HHV8 negative renal transplant recipients became seropositive after surgery.^7^ ^,^ ^8^ Alone, HHV8 infection appears to be insufficient to cause KS and development of the disease relies upon a degree of host immune dysregulation.

Autopsy examination also revealed HHV8 positive intravascular lymphoma in both cases. Several lymphoproliferative conditions, including primary effusion lymphoma, multicentric Castleman disease, HHV8 positive diffuse large B-cell lymphoma and germinotrophic lymphoproliferative disorder, have all been shown to develop as a result of HHV8 infection.^7^ ^,^ ^9^ These HHV8-driven lymphoproliferative disorders are rarely reported and classification can be challenging. Our classification of 'intravascular HHV8 positive DLBCL, NOS' corresponds to the recently introduced entity described by The WHO^10^ as a monoclonal proliferation of HHV8-infected lymphoid cells resembling plasmablasts. Unlike either of our cases, these are almost always associated with Castleman's disease, often in the setting of HIV infection. Most often they are mass-forming or present in a cavity, unlike our intravascular pattern, which is rarely observed. The typical immunophenotype includes CD79a and CD138 negativity, and strong expression of IgM with lambda light chain restriction; unfortunately, it was not possible to assess for light chain restriction in either of our post-mortem cases.

One could argue that these intravascular lymphomas might be better classified as HHV8-associated 'plasmablastic lymphoma'. In a recent publication following the 2015 Workshop of the Society for Hematopathology/ European Association for Hematopathology, which reviewed HHV8 positive lymphoproliferative disorders including plasmablastic and plasma cell neoplasms, it was concluded that the lesions grouped under the term “plasmablastic lymphoma” are heterogeneous with differences that may be related to the immunodeficiency state of the patient (i.e. HIV and post-transplant lymphoproliferative disorder).^11^ Plasmablastic lymphomas occurring in the setting of iatrogenic immunosuppression were defined as being characterized by phenotypic evidence of plasmacytic differentiation with variable loss of B-cell markers, including CD20 and PAX5, and positive expression of plasma cell markers. This is a similar immunoprofile to both cases described in this report except that all seven previously reported cases involved a mass in various anatomical sites, whereas in both our cases the lymphoproliferative lesions were purely intravascular and found in multiple organs.

In 2014, Crane et al.^12^ reported an unusual presentation of HHV-8 positive intravascular lymphoma in a HIV-positive 59-year-old man. Histology demonstrated atypical intravascular lymphoid cells that lacked strong expression of most B-cell markers but were positive for MUM-1 and showed partial expression of several T-cell markers. HHV-8 and an *in situ* hybridization for EBV were both positive in the neoplastic cells. Interestingly, they also described extensive organizing thrombi within the small vessels, which surrounded and obscured the neoplastic cells. The lymphoma was thought to represent an entirely intravascular form of primary effusion lymphoma. Similar to both of our case reports, the disease had a rapidly progressive and fatal course. In a separate case report, Ferry et al.^13^ described the autopsy of a HIV-positive patient, also aged 59 years. At autopsy they found an intravascular large B-cell lymphoma that was positive for MUM-1, HHV8 and IgM lambda, and negative for CD20 and EBER. The lungs, heart, kidney, liver and spleen were involved. The patient was diagnosed with HHV8-positive, EBV-positive large B-cell lymphoma with monotypic IgM lambda expression. Our cases share a number of similarities with both of these reports. Our patients were also immunocompromised (though due to renal transplantation rather than HIV infection) and the lymphoma was systemic and entirely intravascular, with a similar immunophenotype; however, both our cases were negative with EBV and were not associated with thrombus formation. Overall, it is felt that the classification of 'intravascular HHV8 positive DLBCL, NOS' most accurately describes the diseases observed in both our reported cases.

Diagnosis of intravascular lymphoma is challenging and it is not uncommon for this diagnosis to be made post-mortem. In ‘Case 1’ the patient presented with sepsis; we suggest these symptoms were due to the lymphoproliferative disorder. In fact, in the largest published series of 38 cases of intravascular lymphoma, Ferreri et al.^14^ demonstrated that the majority of patients usually presented with non-specific symptoms associated with a marked deterioration in functional status. Fifty-seven percent of subjects had B-symptoms. Sepsis remains the main differential diagnosis in patients who are immunosuppressed presenting with non-specific symptoms. In ‘Case 1’ a full septic screen was negative.

The question of whether to screen organ donors for HHV8 infection is unresolved. Prevalence of HHV8 seropositivity varies between geographic areas and ethnic groups. The rate of HHV8 associated complication may vary depending on the organ transplanted. One case series demonstrated less that 5% of renal transplant recipients developed HHV8 symptomatic infection or HHV8 driven malignancy compared to recipients of heart transplant (50%) and liver transplant (17%).^8^ HHV8 serological screening in the post-transplant setting may be of significant clinical utility. The use of molecular diagnostic techniques, particularly PCR, can be employed to screen for HHV8 infection, though this is an expensive and lengthy process that may not be appropriate in an emergency setting.^15^ Perhaps a more efficient screening method might be testing for antibodies to HHV8, though these methods are variably specific in identifying viral seroprevalence.^16^

## CONCLUSION

HHV8 associated lymphoproliferative disorders are rare and diagnostically challenging entities. HHV8 driven neoplasms are commonly seen in the setting of HIV infection and in HHV8 endemic areas such as the Mediterranean and Sub-Saharan Africa. Considering that the at-risk population is in generally poor resource countries, the low numbers of HHV8 driven lymphoproliferative disorders may not be a true representation of the global disease burden.^.4^ There may be under-reporting and/or misdiagnosis because of limitations on resources as well as expertise in the key geographical areas with the highest prevalence of HIV and HHV8. It is important for clinicians to consider such rarities in immunocompromised patients presenting with non-specific symptoms similar to those of sepsis, particularly if thorough investigation for infection yields negative results. Increased awareness among clinicians and pathologists of HHV8 driven neoplasms will undoubtedly help build upon the limited data that is currently available.
